# MSCs feeder layers induce SMG self-organization and branching morphogenesis

**DOI:** 10.1371/journal.pone.0176453

**Published:** 2017-04-27

**Authors:** Mahmoud Farahat, Gulsan Ara Sathi, Emilio Satoshi Hara, Hiroaki Taketa, Takuo Kuboki, Takuya Matsumoto

**Affiliations:** 1 Department of Biomaterials, Okayama University, Okayama, Japan; 2 Department of Oral Rehabilitation and Regenerative Medicine, Okayama University, Okayama, Japan; 3 Department of Bio-Systems Engineering, Graduate School of Science and Engineering, Yamagata University, Yamagata, Japan; 4 Center for the Development of Medical and Health Care Education, Okayama University, Okayama, Japan; Kyoto Daigaku, JAPAN

## Abstract

Dysfunction of salivary glands leads to several oral health problems, including dental caries, mastication and swallowing dysfunctions and multiple oral infections. Conventional treatments for such condition fell short of providing satisfying therapeutic results. Recent advances in organ regeneration therapy which utilize tissue stem cells to fabricate bioengineered 3D organ buds, have introduced a promising therapeutic tool for full functional organ regeneration. However, finding a sustainable and easily accessible cell source for such approaches is still challenging, especially in case of severely atrophied tissues such as irradiated salivary glands. In response to this, we hypothesized that bone marrow derived mesenchymal stem cells (MSCs) could be used as feeder cells to induce salivary epithelial tissues/cells branching. Indeed, in 2D cultures, MSCs supported branching of embryonic submandibular salivary gland (SMG) epithelium. Interestingly, this enhancing effect was dependent on the initial number of MSC feeder cells. In addition, MSCs supported the self-assembly of SMG epithelial progenitor cells into well-patterned and branched 3D salivary organoids. Therefore, these findings propose MSCs as a valuable candidate cell source for induced SMG epithelial branching, which can potentially be applied in future methods for SMG regeneration approaches.

## Introduction

Saliva plays a key role in maintaining oral health and homeostasis through participating in various natural processes such as mastication, digestion, swallowing as well as protection against dental caries and other types of oral infections [1]. Salivary glands are ectodermal organs that develop through the reciprocal interactions of two distinct tissues, epithelium and mesenchyme. This spatio-temporal and dynamic epithelial—mesenchymal interaction orchestrates glandular cell migration, proliferation and differentiation [2]. Dysfunction of salivary glands which can occur due to several factors such as Sjögren's syndrome, radiation therapy of head and neck tumors and natural aging process, results in a critical health condition known as dry mouth or xerostomia [3].

A variety of therapeutic approaches have been used for treatment of xerostomia including use of artificial saliva substitutes and other drugs to induce salivary flow [4]. However, the limited success of such approaches specially for patients with massive salivary tissue atrophy have indicated the importance of introducing novel therapeutic methods for salivary gland replacement. In this context, recent attempts of salivary gland regeneration have been brought under research spotlights. For example, Ogawa et al. succeeded in fabricating a functional salivary gland from an organ germ utilizing epithelial and mesenchymal stem/progenitor cells derived from embryonic salivary glands [5]. However, despite such remarkable progress, the capability of these methods to generate salivary gland tissue of sufficient size, and resembling that induced by natural gland organogenesis has not been achieved. In addition, since most of these approaches utilize salivary gland stem/progenitor cells, shortage of such cell source is one of the major concerns, especially in cases of dramatic salivary gland dysfunction, such as after irradiation therapy, in which the surviving salivary progenitor cells lose their capability to differentiate into acinar cells [6].

Consequently, attempts to regenerate functional salivary glands have to face a challenge to find a cell source for replacing the damaged tissues and cells [7]. In response to these challenges, we propose that MSCs [8] could be applied as a suitable mesenchymal feeder-cell source for inducing salivary epithelial morphogenesis. MSCs are considered as excellent candidates for cell-based tissue engineering approaches due to their well-known characteristics of unlimited self-renewal capacity and potential to differentiate into multiple lineages [9]. In addition, MSCs have shown the capacity to be used as feeder layers for epithelial cells such as pancreatic islets and corneal epithelial cell sheets [10,11]. Furthermore, MSCs can be easily extracted from the bone marrow cavities of both embryonic and adult tissues with simple and well-established protocols. Concomitantly, MSCs exhibit a powerful capacity for in vitro culture expansion [12]. In addition, recently MSCs donor banks had been already established to serve as stock sources for high-quality donor cells for therapeutic purposes.

In this study, we attempted to show promising results for the use MSCs as feeder layer cells supporting branching of primary SMG epithelium tissues in 2D cultures. Additionally, our data suggest that MSCs supported the self-assembly of primary SMG epithelial cells and its subsequent branching into multiple branched buds in 3D organotypic culture.

## Materials and methods

### Whole SMG dissection, mesenchyme-free SMG epithelial rudiments and cell isolation

ICR female mice (Charles River Laboratories, Japan) were purchased, delivered at E12 and kept in sterile ventilated cages with access to food and water *ad libitum*. At E13, mice were sacrificed by CO_2_ asphyxiation and used in experiments. Mouse care and animal handling were performed in accordance to “Guidelines for Animal Experiments at Okayama University” and all experimental protocols were approved by the Animal Care and Use Committee Okayama University (OKU-2013039 and OKU-2015540).

Embryos were harvested at embryonic day E13 then SMGs were isolated from embryos under dissecting microscope (STZ- 40Tba Shimadzu, Japan). Isolated SMGs were treated with 4 U/ml dispase 1 (Roche, Switzerland) for 5 min at room temperature and then mechanically separated into epithelial and mesenchymal tissues using fine forceps. For single cell isolation, whole SMG tissues, isolated mesenchymal tissues and mesenchyme-free epithelial rudiments were treated twice with 100 U/ml collagenase I (Worthington, NJ) in PBS solution without calcium or magnesium at 37°C for 10 min on the shaker, then treated with 0.25% trypsin-EDTA (Sigma-Aldrich, MO) for 5 min at 37°C on a rotary shaker. After enzymatic treatment, tissues were re-suspended in Dulbecco’s Modified Eagle’s Medium (DMEM, Wako pure chemical, Japan) supplemented with 1% Penicillin/Streptomycin (PS, Nacalai Tesque, Japan) (DMEM/F-12/PS) containing 10 μg/ml DNase I (Roche). Finally, tissues were dissociated into single cells by gentle pipetting followed by filtering through 70 μm Nylon filter (BD Falcon, NC). For use in 2D culture, dissociated mesenchymal cells were re-suspended in DMEM/F-12/PS, counted and then seeded in 96-well plates to yield an initial cell concentration of 7.5 x 10^4^ cells per well for 24 h. For 3D cultures, cell concentration of the dissociated whole glands or epithelial rudiments was measured using hemocytometer and obtained cells were aliquoted into grease-coated 1.5 ml tubes to yield 4 x 10^4^ cells per tube.

### MSCs isolation and culture

Bone marrow derived MSCs were isolated from bone marrow of BALB/C1 mice (Shimizu Laboratory, Japan) as previously descried [13]. Briefly, MSCs were generated from tibia and femur bone marrow of 8 week-old mice. Cells were cultured in basic medium alpha minimal essential medium (α-MEM, Wako pure chemical) supplemented with 10% heat-inactivated fetal bovine serum (FBS, Invitrogen, CA) and 1% PS (Nacalai Tesque). Non-adherent cells were removed after 24 h, and adherent cells were harvested and re-cultured in 75 cm^2^ flasks for 3–4 passages before using. Medium was replaced every 3 days. MSCs were characterized by immunostaining analysis using monoclonal antibodies (Sca-1, CD44 and CD105). MSCs were then enzymatically collected from culture flasks 24 h prior to experiment day and washed carefully with PBS. Cells were re-suspended in DMEM/F-12/PS, counted using hemocytometer and then seeded in 96-well plates to yield an initial cell concentration of either 1 x 10^4^, 5 x 10^4^, 7.5 x 10^4^ or 1 x 10^5^ cells per well for 24 h. For experiments in which epithelial/MSCs combined 3D cell aggregates were used, dissociated epithelial cells and MSCs were counted separately and mixed thoroughly together with different cell ratios: 2:1, 1:1 or 2:1 (epithelial cells: MSCs). Epithelial/MSCs cell mixture were then aliquoted into grease-coated 1.5 ml tubes to yield 2 x 10^4^, 3 x 10^4^ or 4 x 10^4^ cells per tube for further use.

### NIH/3T3 cell culture and seeding

NIH/3T3 fibroblast cell line was cultured in a mixture of DMEM/F-12/PS and 10% newborn calf serum (NBCS) (Life Technologies, NY) in a humidified incubator under 5% CO_2_ in air atmosphere. Cells were enzymatically collected 24 h prior to experiment day and washed carefully with PBS. Cells were re-suspended in DMEM/F-12/PS, counted and then seeded in 96-well plates to yield an initial cell concentration of 7.5 x 10^4^ cells per well for 24 h.

### MEF cell culture and seeding

For MEF isolation, we used previously reported method [14]. Briefly, E13 mice embryos were isolated and washed with PBS and heads and visceral tissues were removed. The remaining bodies were washed, minced, transferred into 0.5% trypsin-EDTA (3 ml/embryo), and incubated at 37°C for 40 min. After trypsinization, tissues were re-suspended in an equal amount of medium (DMEM containing 10% FBS) followed by pipetting to complete tissue dissociation. Next, the supernatant cell mixture was transferred into a new tube. Cells were collected by centrifugation (200 g for 5 min at 4°C) and resuspended in fresh medium and cultured on 100 mm dishes at 37°C with 5% CO_2_. For using in experiment, cells were enzymatically collected 24 h prior to experiment day and washed carefully with PBS. Cells were re-suspended in DMEM/F-12/PS, counted and then seeded in 96-well plate to yield an initial cell concentration of 7.5 x 10^4^ cells per well for 24 h.

### 2D SMG epithelial rudiment culture

After 24 h of seeding, the formed feeder cell layers (SMG mesenchyme, MSCs, NIH/3T3 or MEF) were examined under microscope. Then, isolated epithelial rudiments were seeded on top of each formed feeder layer and incubated for 72 h in DMEM/F-12/PS medium. Branching of epithelial rudiments was monitored under microscope (TE-2000, Nikon, Japan) and quantified as the fold-change in number of buds at each time point divided by number of buds at 0 h. Each experiment was repeated at least 5 times. Bud number quantification in each experiment was carried out by four independent observers using Image J software (NIH, MD) and the average of their quantification of the multiple experiments was used in comparison.

### Three-dimensional 3D cell aggregate culture

For 3D cell spheroid culture, the aliquoted cells (dissociated whole SMG cells, dissociated epithelial cells and epithelial/MSCs aggregates) were separately pelleted by centrifugation at 900 g for 3 min, and the obtained cell pellets were seeded using a 20 μl pipette inside a 40 μl drop of growth factor reduced Matrigel (BD Biosciences, MA) diluted 1:1 with DMEM/F-12/PS medium and incubated at 37°C for 72 h. Growth factors; EGF 20 ng/ml and FGF7 100 ng/ml (both from R&D systems, MN, USA) were selectively supplemented to the seeded aggregates in accordance with each experiment protocol.

### Quantification of self-assembly process of epithelium/mesenchymal and epithelium/MSCs cell aggregates

Self-assembly of epithelium/MSCs, whole SMG and only epithelial cell aggregates were observed under microscope (TE-2000). Bright field and immunostained images of each aggregate were used to quantify buds’ number per aggregate at each time point using Image J software. Each experiment was repeated at least 5 times and the average of multiple experiments was used in comparison. Cell aggregates were incubated at 37°C supplemented with 5% CO_2_ and 95% humidified air in a hand-made environmental chamber fitted onto an inverted microscope (Eclipse Ti, Nikon). Images were acquired using bright field optics through 10x objectives once every 15 min for 72 h and were assembled into movies in AVI format using Image J software. Changes in aggregate size and shape were observed by performing serial measurements of the aggregate area from 0 h. Change in aggregate circularity (C) was measured as 4πA/ L^2^, where A is the aggregate projected area and L is the contour line length.

### Immunofluorescent staining

Samples were fixed with 4% paraformaldehyde for 20 min at room temperature and washed with PBS containing 1% bovine serum albumin (BSA) (Nacalai teasque) and 0.1% Triton X-100 (Sigma-Aldrich) (PBSX). Samples were then blocked with blocking solution (One Histo, Nacalai teasque) followed by incubation with primary antibodies diluted into PBSX. Used primary antibody were: mouse anti-CD105 (1:300, eBioscience, CA), goat anti-Sca-1 (1:200, eBioscience), rabbit polyclonal anti-CD44 (1:300, Abcam, UK), rabbit anti collagen IV (1:250, Abcam), and fluorescein isothiocyanate (FITC)-conjugated lectin from *Arachis hypogaea* (peanut), (PNA, 1:200, Sigma-Aldrich). For nucleus staining, DAPI (Life Technologies, NY) was used. Antibody bindings were detected using Alexa fluor-conjugated secondary antibodies (Life Technologies) and images were obtained using a confocal laser scanning microscope (C1, Nikon).

### Statistical analysis

Quantification of the buds were done and mean values with standard deviation were then calculated. Statistical significance was evaluated using one-way analysis of variance (ANOVA) post hoc test with Scheffe’s F test when needed with p values included in the figure legends.

## Results

### MSCs function as a novel feeder layer for salivary epithelial morphogenesis in 2D culture

**Fig 1:**
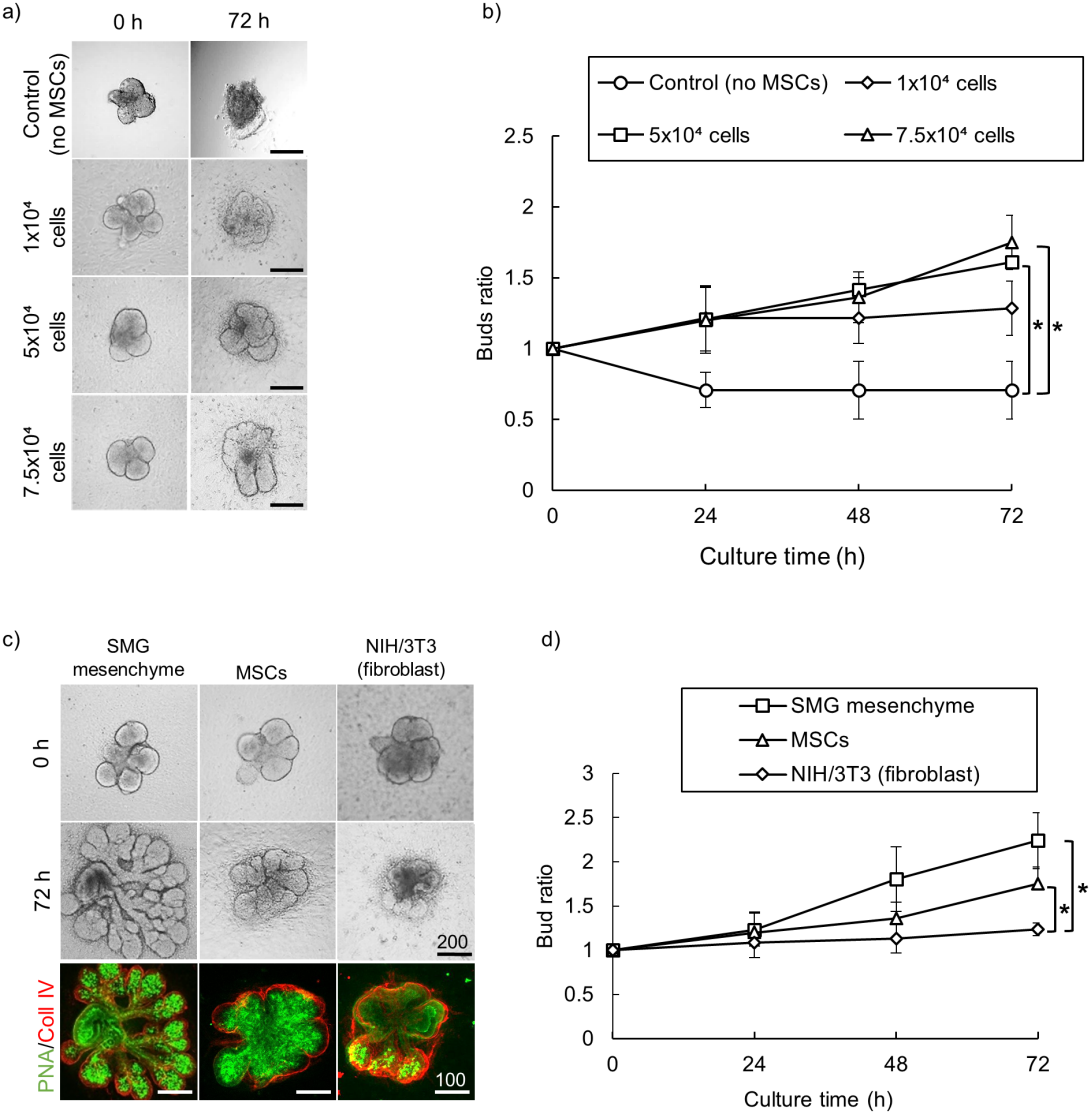
MSC feeder layer induces salivary epithelial morphogenesis in 2D culture. (a) Evaluation of branching morphogenesis of isolated E13 epithelial rudiments cultured on top of MSC feeder layers with different initial cell concentrations. (b) Quantification analysis of epithelial branching showing that MSC feeder layers significantly induced epithelial bud formation in cell concentration-dependent manner with a maximum increase up to 1.6-fold at an initial cell concentration of 7.5 x 10^4^ cells per well. Branching was quantified at each time point by counting the number of buds per gland divided by number of buds at 0 h. (* p ˂ 0.01, One-way ANOVA, Scheffé's F post-hoc test. Scale bar: 200 μm). (c) Comparative analysis of MSCs feeding effect compared to native SMG mesenchyme cell layers (positive control) and NIH/3T3 fibroblast cell line (negative control). Cell concentration = 7.5 x 10^4^ cells per well. Lower panel show cultured epithelial buds stained with specific epithelial marker PNA and counterstained with collagen type IV as a specific basement membrane marker to delineate borders of each of the branched buds. (Scale bar: 200 μm). (d) Quantification analysis of epithelial morphogenesis showing that MSCs feeder layers induced higher epithelial bud branching compared to NIH/3T3 cell line (1.6 and 1.2-fold increase respectively), though showing less, while not significant, effect than native SMG mesenchyme. (* p ˂0.01, One-way ANOVA, Scheffé's F post-hoc test.)

Since MSCs have shown the capacity to be used as feeder layers for epithelial cells such as pancreatic islets and corneal epithelial cell sheets [10,11], we hypothesized that MSCs could be used successfully as feeder cells for primary salivary epithelium morphogenesis. MSCs were isolated from the bone marrow of Balb/C mice as described earlier. The nature of isolated cells as MSCs was confirmed via immune staining for CD105, C-kit and CD44 (S1 Fig). MSCs were seeded in 96 well-plate with different initial cell concentrations of (1 x 10^4^, 5 x 10^4^, 7.5 x 10^4^ and 1 x 10^5^ cells per well). Next, salivary epithelial rudiments were isolated and cultured on top of the formed MSCs layers. Interestingly, we found that MSCs feeder layers supported epithelial morphogenesis and induced bud formation of epithelial rudiments up to 1.6-fold of buds’ ratio in a cell concentration-dependent manner up to 7.5 x 10^4^ cells per well (Fig 1a and 1b). A higher concentration of MSCs (1 x 10^5^ cells per well) promoted initial epithelial growth. However, most of the seeded samples collapsed afterwards and detached from the tissue culture plates.

Next, we sought to compare the feeding effect of MSCs to the native SMG mesenchymal cells as positive controls, and NIH/3T3 fibroblasts, a standard fibroblast feeder layer [15], as negative control. We seeded each cell type at an initial cell concentration of 7.5 x 10^4^. As expected, native SMG mesenchymal induced the highest epithelial branching with 2.3-fold increase in buds’ ratio after 72 h culture. However, our results showed that although MSCs induced less branching than SMG mesenchyme, they induced higher bud branching than NIH/3T3 cells (Fig 1c and 1d). Also, we tested MSCs feeding effect against mouse embryonic fibroblast (MEF), a standard primary feeder cells for stem cell culture [14]. Interestingly, both cell types showed similar results in inducing branching of isolated epithelial rudiments (S2 Fig). Taken together, these data suggest that MSCs can be used effectively as a novel feeder layers for salivary epithelial tissue morphogenesis in 2D culture condition.

### MSCs can induce primary epithelial cells self-assembly and branching in 3D culture

**Fig 2:**
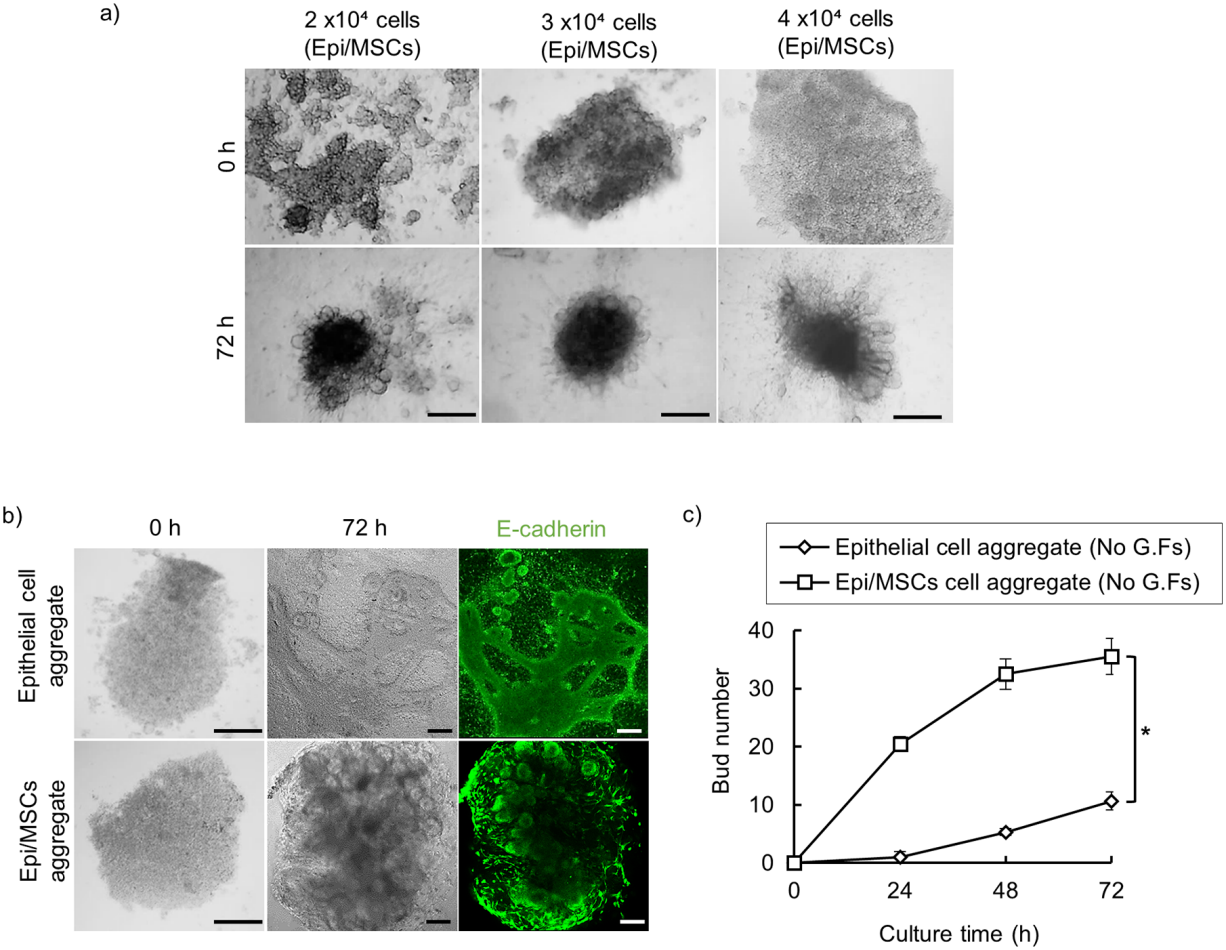
Generation and optimization of *in vitro* 3D epithelial cells/MSCs spheroids. (a) Examination of possible cell combinations with different initial cell concentrations. (b) Evaluation of MSC effect in inducing self-organization of salivary epithelial cells. In absence of exogenous growth factors, epithelial cell aggregates without MSCs, self-assembled into amorphous aggregates with very few buds could be observed; whereas epithelial cells/MSCs aggregates self-organized into 3D cell spheroids. (c) Bud formation in the developing epithelial cells/MSCs aggregates. Aggregates were stained with an antibody to the epithelial-specific marker, E cadherin (green), (Scale bar: 100 μm) Left; bright field image shows the elongating structure in cell aggregate (d) Quantification analysis of bud number per aggregate. Epithelial cells/MSCs aggregates showed multiple bud formation while only epithelial cell aggregates failed to branch into well-developed organoids in absence of growth factor supplementation (* p ˂ 0.01, One-way ANOVA, Scheffé's F post-hoc test. Scale bar: 200 μm).

Next, we hypothesized that this inductive capacity of MSCs could be extended into 3D epithelial cell culture model. For this purpose, we developed a 3D culture system to fabricate an epithelial cell/MSC combined organoid in a biomimetic environment. To identify the optimal condition for constructing the combined cell organoid, we explored different initial cell concentrations and cell-cell ratios within the 3D aggregates. We initially used 1 x 10^4^ MSCs and 1 x 10^4^ epithelial cells (1:1 cell ratio) to make 2 x 10^4^ cells combined cell spheroids. Aggregates were initially supplemented with EGF 20 ng/ml and FGF7 100 ng/ml as previously reported [16]. Results showed that under these culture conditions, natural self-organization of the aggregated cells into 3D spheroids could be slightly reproduced with only a few number of buds formed. Thus, next we tried higher concentrations for cell spheroid (3 x 10^4^ and 4 x 10^4^) with different epithelial/MSCs ratios (2:1 and 1:2). Interestingly, we found that cell concentration of 4 x 10^4^ cells incorporating 2:1 epithelial cells/MSCs ratio adopted more compacted and circular morphology, with more formed buds at the peripheries of each aggregate (Fig 2a).

### MSC itself induce epithelial cells assembly and branching

To address whether MSCs can directly induce epithelial cells self-organization independent of exogenous stimulation with growth factors, we then seeded epithelial cells/MSCs aggregate (4 x 10^4^ cells with 2:1 epithelial cells: MSCs ratio) in a 40 μl drop of growth factor reduced Matrigel diluted with DMEM/F12 medium without any growth factor supplementation, and compared with only epithelial cell aggregate with the same cell concentration (4 x 10^4^ cells) (Fig 2b). Interestingly, we found that epithelial cells/MSCs aggregates self-organized into well-shaped 3D cell spheroids with multiple epithelial bud formation. To confirm the epithelial bud branching, we performed immunostaining of the developing epithelial cells/MSCs aggregate for a specific epithelial marker E-cadherin to show organized spheroids with a group of E-cadherin-positive epithelial buds aligned at the peripheral parts (Fig 2c). On contrary, only epithelial cell aggregate could only self-assemble into loose aggregates that failed to subsequently branch (Fig 2d).

### Epithelial cells-MSCs spheroids have unique cellular structure compared to whole SMG cell spheroids

**Fig 3:**
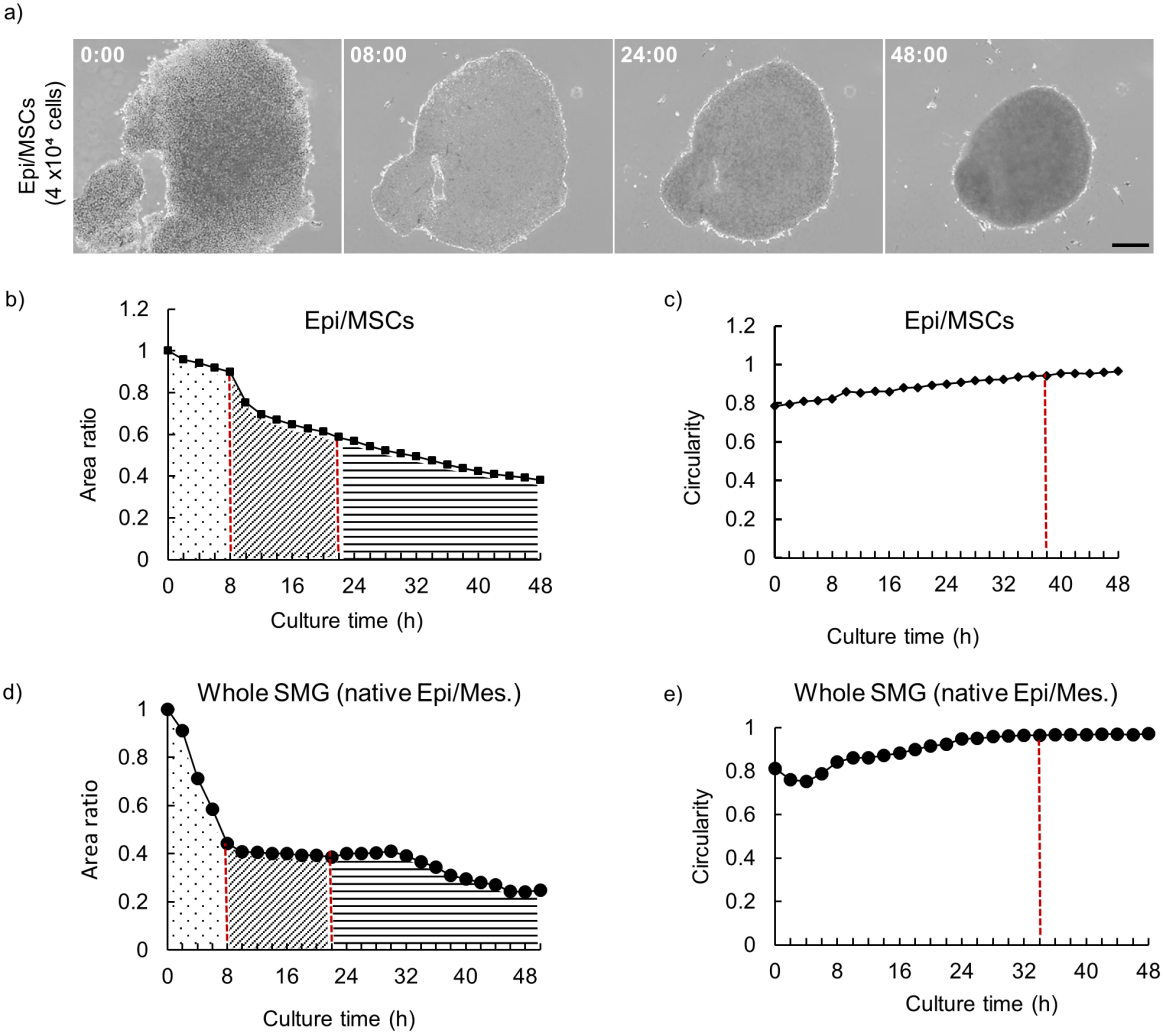
Time-lapse analysis of self-driven aggregate development. (a) Frames from time-lapse S1 Movie showing self-driven condensation of epithelial cells/MSCs aggregate into 3D cell spheroid. Time represented in hours. Scale bar: 100 μm. (b) Examination of size changes during spheroid development. The aggregate projected area rapidly decreased in the first 24 hours of culture then showed steady and gradual decline. (c) Structural analysis of dynamic morphological changes during 3D spheroid development. Red dotted line marks the closest point of the aggregate as a circle (balance state). The source movie is S1 Movie. Aggregate circularity C was defined as 4πA divided by L^2^, where A is the aggregate projected area and is L^2^ the square of the contour line length. (d) Comparative analysis of size changes during whole SMG (native SMG epithelium and mesenchyme) spheroid development. Notably, the aggregate projected area decreased sharply in the first 8hours of culture then showed steady and gradual decline. (e) Comparative analysis of whole SMG aggregate morphological (circularity) changes during 3D spheroid development.

To further dissect the effect of MSCs in self-organization of epithelial cells, we thought to analyze the underlying dynamic cellular interactions during spheroid development. For this purpose, we used time-lapse imaging to track and analyze the self-assembly of epithelial cells/MSCs aggregates into their tissue-like 3D patterns. The self-assembly process has been reported to be constituted of 3 stages: migratory stage, compaction stage and bud branching stage. As shown in Supplementary S1 Movie, and depicted in Fig 3a, epithelial cells/MSCs aggregate self-assembly proceeded in a comparable ordered multi-step process. Time-course analysis of the changes in aggregate size (area) and shape (circularity) during tissue self-assembling showed that the total cell aggregate area of epithelial cells/MSCs spheroids slightly decreased in the initial migratory stage (0–8 h), while this decrease in cell aggregate area was remarkable in the case of epithelial cells/native mesenchymal cells aggregates (Fig 3b and 3d; black dotted area). In the subsequent stage of cellular compaction (8–24 h), shrinkage of epithelial cells/MSCs spheroids resulted in further condensation of the cell aggregate to approximately 40% shrinkage from the initial aggregate area. In the case of epithelial cells/native mesenchymal cells aggregates, this shrinkage was more evident, reaching the lowest projected area (of approximately 80% shrinkage from the initial aggregate area) at 16 h (Fig 3b and 3d; diagonal striped area).

The final step (24–48 h) (Fig 3b and 3d; horizontal striped area); high compaction occurs shaping the aggregate into more circular form with aggregate circularity (the structural indicator of how close the aggregate shape is to that of a complete circle) gradually increases reaching a nearly constant value of 0.96 at *t* ≤ 40 h (Fig 3c). This observation of the aggregate spherical transformation is generally associated with the aggregate reaching an equilibrium state between the increasing condensation force and the adhesion strength of cell-extracellular matrix environment [17]. Notably, at such equilibrium state, aggregated cells minimize the surface free energy of their system and thus, it has been generally proposed that tissues develop via a succession of equilibrium states in which the sum of the mechanical forces is in balance [18].

**Fig 4:**
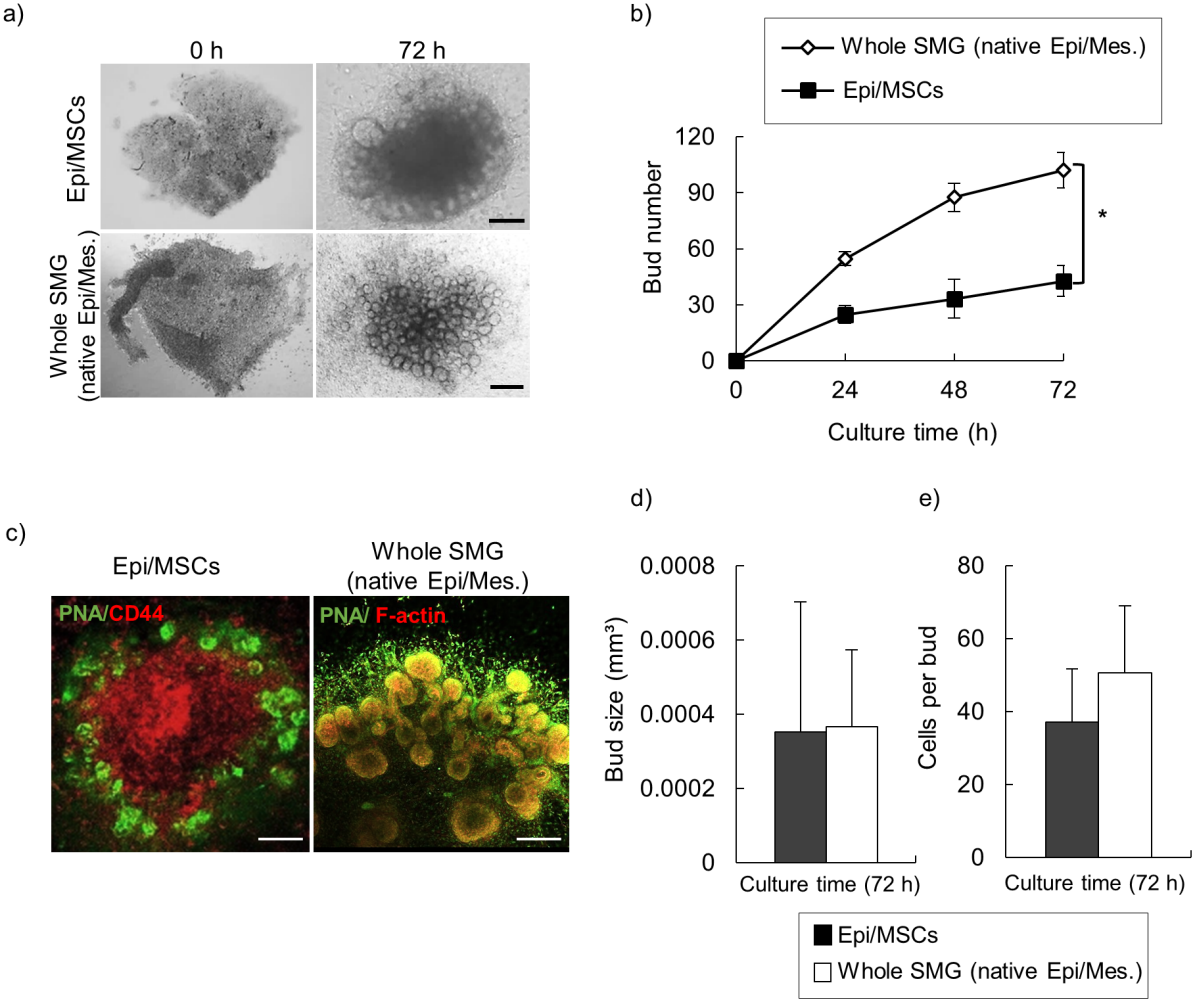
Morphological and structural characteristics of epithelial cell/MSCs and whole SMG cell spheroids. (a) Morphological evaluation of both epithelial cells/MSCs spheroids and whole SMG cell. Scale bars: 100 μm and 200 μm, respectively. (b) Quantitative analysis of bud’s number per spheroid. Whole SMG spheroids show significantly higher number of buds compared to epithelial cells/MSCs spheroids (* p ˂ 0.01, One-way ANOVA, Scheffé's F post-hoc test). (c) Structural analysis of cell spheroids. Left; immunofluorescence analysis of epithelial cells/MSC spheroid using antibody for specific epithelial marker PNA (green) and MSCs marker CD44 (red). CD44-positive MSCs occupied the center of the cell spheroid encircled by PNA-positive epithelial buds aligned at outer layer. Scattered CD44-positive MSCs at the most-outer layer surrounded the formed epithelial buds. (Scale bar: 100 μm). Right; whole SMG cell aggregate stained with an antibody to PNA (green), and counterstained for F-actin with rhodamine phalloidin (red) (Scale bar: 100 μm). (d) Bud size quantification in both types of spheroids. (d) Quantification analysis of cell number per bud in both types of spheroids. Buds in SMG spheroids show slight increase in cell number per bud compared to buds formed in epithelial cells/MSCs spheroids.

To further understand the effect of these unique cellular dynamics on the formed epithelial buds, we compared between epithelial cell/MSCs spheroid and the dissociated whole SMG spheroid. As expected, we found that number of formed buds in the dissociated whole SMG cell aggregate was significantly higher than those formed in the epithelial cells/MSCs spheroid (Fig 4a and 4b). Interestingly, immunofluorescence analysis of epithelial cells/MSC spheroid using antibody for specific epithelial marker (PNA) and MSCs marker (CD44) showed a distinct cell patterning in which the formed epithelial buds were aligned at outer layer of the formed aggregate surrounding an inner most layer (core layer) of the highly compacted MSCs (Fig 3c, left). On Contrary, in whole SMG cell aggregate, the formed epithelial bud resided at the center surrounded by a cloud of scattered cells (Fig 3c, right). Next we hypothesized that the different level of cell aggregate compression between whole SMG and epithelial cells/MSCs spheroids will reflect on the sizes of the formed buds and the number of cells forming each bud. However, our results didn’t show significant difference regarding the size of buds or the number cells per buds in both cell aggregates types (Fig 4c and 4d).

## Discussion

Submandibular salivary gland (SMG) has long been the model of choice for studying branching morphogenesis, the key developmental process for many glandular organs including lung, kidney, mammary and salivary glands [19,20]. Intensive studies identified multiple growth factors and several processes such as cell-cell and cell-matrix interactions to be essential for this process [21]. In particular, epithelial-mesenchymal interactions, the reciprocal communications between epithelium and its mesenchymal counterpart with its multicellular cell population, proved to be the cornerstone in glandular branching morphogenesis [22]. The nature of epithelial-mesenchymal interaction in SMG in particular, draws lots of attention because of the specific nature of SMG mesenchyme. Early reports showed that epithelium isolated from SMG failed to branch when it was combined with mesenchyme from other sources whether non-glandular mesenchyme or from other glandular tissues [23, 24].

On the contrary, SMG embryonic mesenchyme was shown to induce epithelium from other glandular sources to develop into a branched gland that morphologically resembles natural salivary gland [25]. These findings therefore, demonstrated a specific need of SMG mesenchyme for proper morphogenesis and development of SMG. This fact posed a challenge to find a suitable and easily accessible cell source for salivary gland replacement therapy. In this context, here we report promising results for the use of bone marrow derived MSCs as feeder cells to promote branching of primary salivary epithelial cells. Our results showed that in 2D cultures, isolated salivary epithelial tissues co-cultured on top of MSCs layers were able not only to maintain their viability but also underwent a successful branching. This feeder effect depended on the initial density of the seeded MSCs. In general, a typical feeder layer supports the growth of the co-cultured cells through secretion of essential growth factors and/or providing adhesion molecules and ECM components for cell attachment. Our 2D culture results showed that MSC feeder layer promoted epithelial branching, however to a slightly lower extent compared to that induced by the native SMG mesenchyme feeder layer. Nevertheless, MSCs clearly present a higher induction potential than the conventionally used fibroblasts, as feeder layers. MSCs have been reported to secrete a plethora of growth factors which might account for this feeding effect. Among those previously reported factors, HGF and EGF [13] have been regarded as key promoters in salivary gland branching process [26,27]. EGF was found to promote epithelial cell proliferation and branching in isolated SMG epithelial culture [28,29]. An alternative mechanism might involve other factors such as TGF-β which controls SMG branching morphogenesis through regulating ECM synthesis during SMG development [20]. Moreover, it might be possible that combination of several factors and secreted molecules be collectively responsible for MSCs proposed inducing effect as feeder cells.

Recapitulation of the 3D natural organ structure is believed to be crucial to successfully generate a healthy functional tissue [30]. In this context, a major breakthrough has been achieved recently in regeneration therapy through utilizing the concept of cellular self-organization to successfully reconstitute functional 3D organ buds (organoids) [31–33]. Self-organization in biological systems refers to the ability of these systems to spontaneously form an ordered structural pattern in the absence of external guidance [34]. This natural process had been observed in different types of epithelial cells such as Madin-Darby canine kidney cells, mammary and salivary epithelial cells [16,35,36]. In the current study, we used a previously modified 3D culture technique to combine multicellular aggregate composed of a mixture of dissociated SMG epithelial cells and MSCs in matrigel substrate. Interestingly, MSCs were able to support the self-driven morphogenesis of epithelial cells. Time-lapse analysis of this process showed that epithelial cells/MSCs aggregate development proceeded in a dynamic self-organized pattern resembled the pattern reported previously by primary epithelial cells self-organization [16]. Furthermore, this process showed the three characteristic patterns in self-organizing tissues [37**]**: self-assembly of cells forming one coherent aggregate driven by MSCs, self-patterning of cells by which the multicellular population acquired distinct distribution within each aggregate. Finally, self-driven morphogenesis by which budding and branching can be seen in epithelial cells middle ring layer. We found that the emergence of this ordered cellular structure to be a size sensitive phenomenon in which aggregate self-formation and patterning depended on both the initial total number of cells seeded and more importantly on the cellular ratio between MSCs and epithelial cells to reach a sensitive balance between the number of MSCs capable of encapsulating the epithelial cells and induce subsequent branching and number of epithelial cell sufficient for undergoing branching.

## Conclusion

In the present study, we showed that bone marrow derived MSCs supported branching of isolated epithelial rudiments cultured on top of it, in a 2D culture system. Moreover, in a 3D culture model, a mixed cell aggregate of isolated salivary primary epithelial cells and MSCs showed multiple bud formation. The aggregate formation and branching proceeded in organized process, resembling the appearance of the whole SMG cell aggregate development. Taken together, these data suggest that MSCs could potentially be used as a feeder cells for salivary epithelial tissues/cell branching in 2D and 3D culture conditions, and could potentially be a valuable candidate cell source for SMG regeneration studies.

## Supporting information

S1 Fig Microscopic characterization of isolated MSCs. Cells showed positive staining for CD105, C-kit and CD44.
(PDF)

S2 Fig Comparative analysis of MSCs feeding effect to mouse embryonic fibroblast cells (MEF). Quantification analysis of epithelial morphogenesis showed that MSCs feeder layers induced slightly higher epithelial bud branching compared to MEF.
(PDF)

S3 Fig Bud formation in the developing epithelial cells/MSCs aggregates. Aggregates stained with E cadherin (green), counterstained for F-actin with rhodamine phalloidin (red) (Scale bar: 100 μm)
(PDF)

S1 Movie Epi/MSCs 3D spheroid self-organization. MSCs induced condensation and self-patterning of the heterotypic cell aggregate.
(AVI)
