# Supplementary figures and images for: MSCs feeder layers induce SMG self-organization and branching morphogenesis
Source: PLoS One. 2017 Apr 27;12(4):e0176453. doi: 10.1371/journal.pone.0176453 (PMC5407632; doi:10.1371/journal.pone.0176453)

S1 Fig.

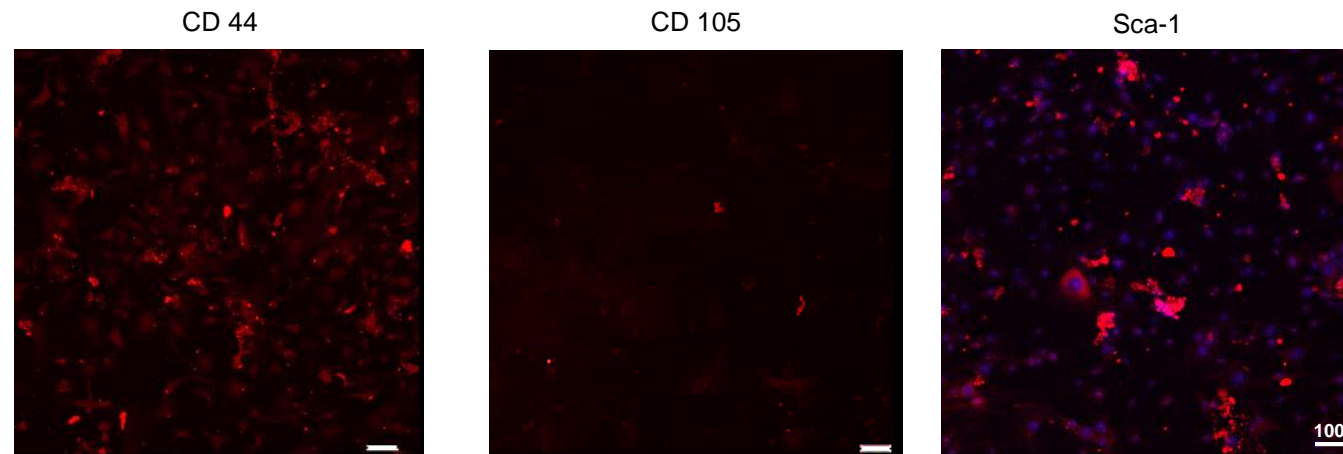

Supplement: S1 Fig — Cells showed positive staining for CD105, C-kit and CD44. (PDF) [file pone.0176453.s001.pdf]

S2 Fig.

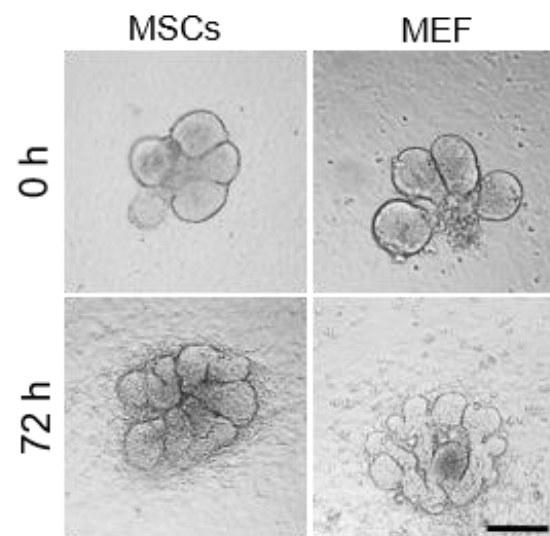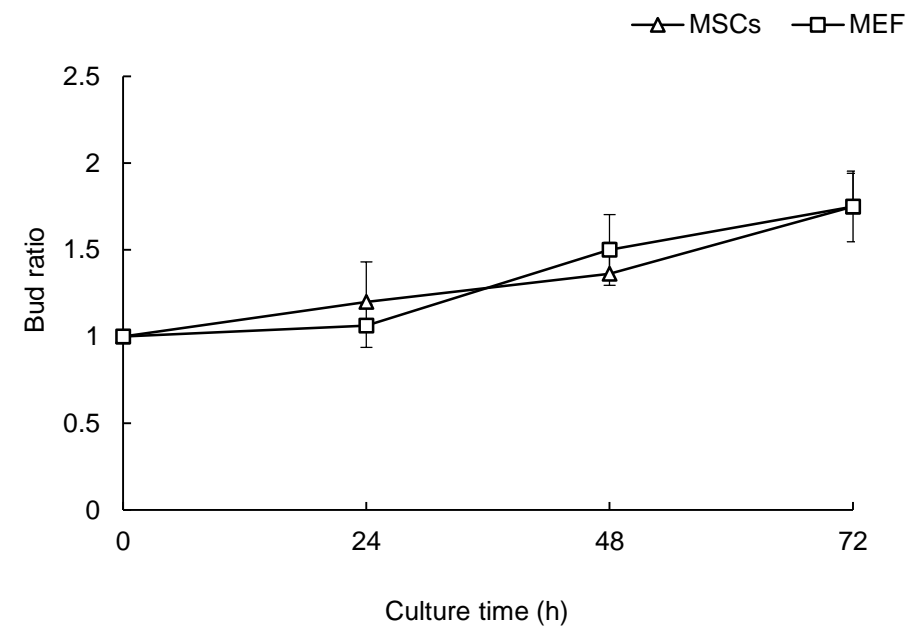

Supplement: S2 Fig — Quantification analysis of epithelial morphogenesis showed that MSCs feeder layers induced slightly higher epithelial bud branching compared to MEF. (PDF) [file pone.0176453.s002.pdf]

S3 Fig.

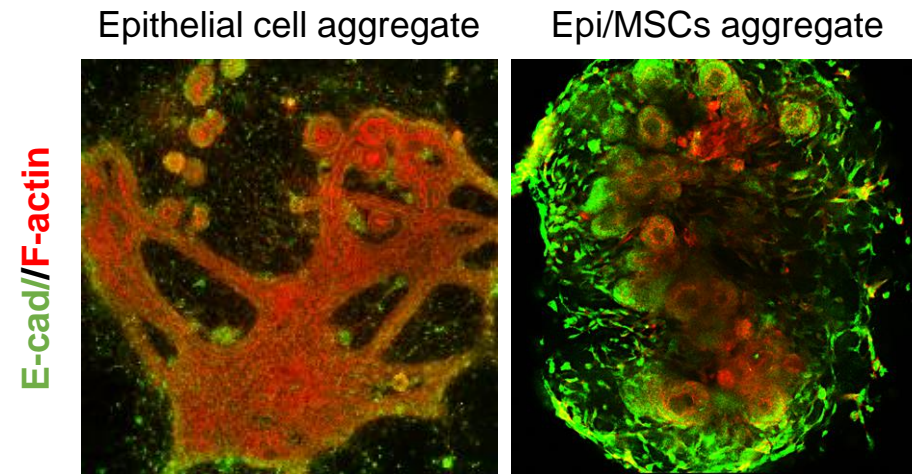

Supplement: S3 Fig — Aggregates stained with E cadherin (green), counterstained for F-actin with rhodamine phalloidin (red) (Scale bar: 100 μm) (PDF) [file pone.0176453.s003.pdf]
